# Menthol–Fatty Acid HDES Boosts In Vitro Oral Bioavailability of Oleanolic Acid via Synergistic Digestive Release and Cellular Absorption

**DOI:** 10.3390/foods15020343

**Published:** 2026-01-17

**Authors:** Qin Zhang, Chenjia Li, Jie Yu, Benyang Li, Chaoxi Zeng

**Affiliations:** 1 Department of Food Science and Technology, College of Food Science and Technology, Hunan Agricultural University, No. 1 Nongda Road, Furong District, Changsha 410128, China; zhangq20001227@163.com (Q.Z.); chenjiali@stu.hunau.edu.cn (C.L.); yuj123@stu.hunau.edu.cn (J.Y.); 2 Xiangya School of Public Health, Central South University, 110 Xiangya Road, Changsha 410078, China; 256901012@csu.edu.cn

**Keywords:** oleanolic acid, hydrophobic natural deep eutectic solvents, simulated digestion in vitro, Caco-2 cells monolayer, bioavailability

## Abstract

To improve the oral bioavailability of oleanolic acid (OA), this study developed a menthol–fatty acid-based hydrophobic deep eutectic solvent (HDES) system. Through a comprehensive evaluation using in vitro simulated digestion and Caco-2 cell transport models, the short-chain HDES was found to increase the apparent in vitro bioavailability index of OA by 9.3-fold compared to conventional ethanol systems, with efficacy showing clear fatty acid chain-length dependence. The mechanism was systematically investigated through spectral characterization and cellular studies, revealing a two-stage enhancement process: during the digestion phase, HDES significantly improved OA bioaccessibility to 14.30% compared to 4.90% with ethanol; during the absorption phase, it markedly increased cellular uptake to 25.79% versus 4.71% with ethanol. Molecular analysis indicated that the optimal hydrophobicity and diffusion properties of HDES contributed to this enhancement. This study reveals a fatty acid chain-length-dependent mechanism in HDES-facilitated OA delivery, providing a tunable strategy for enhancing the absorption of hydrophobic bioactive compounds.

## 1. Introduction

Pentacyclic triterpenoids are a class of secondary metabolites widely present in the plant kingdom [1]. Among them, oleanolic acid (OA) is a representative compound that exhibits a range of significant pharmacological activities, such as anti-inflammatory, hepatoprotective, and hypoglycemic effects [2]. However, most pentacyclic triterpenoids, including OA, generally face a key bottleneck: due to their inherent strong hydrophobicity, their oral bioavailability is low [3,4]. This defect seriously restricts their application potential in the fields of medicine and functional foods, making it difficult for their excellent therapeutic effects to be fully exerted in the body [5]. Therefore, effective delivery strategies are needed to overcome this limitation. Given its typical structure and shared bioavailability constraints, OA was selected in this study to develop delivery approaches applicable to similar lipophilic triterpenoids.

To overcome this challenge, researchers have developed a variety of strategies, currently mainly focusing on two major directions: one is to synthesize OA derivatives with higher water solubility and bioavailability through chemical structure modification, which provides an important chemical basis for new drug development [6]; the second approach is to embed OA in new delivery carrier systems, such as nanoparticles, liposomes, and solid dispersions, aiming to enhance its solubility, stability, and intestinal permeability through physical means [6,7]. However, these existing methods still have obvious limitations: the chemical modification process may be complex and costly and may change the activity of the parent compound or introduce new safety issues; the construction process of carrier delivery systems is often rather complicated, and loading efficiency, physical stability, and the feasibility of large-scale production need to be improved. More importantly, the safety and in vivo metabolic behavior of new carrier materials themselves usually require systematic re-evaluation, which to some extent delays their application process.

In recent years, Natural Deep Eutectic Solvents (NADESs) have attracted extensive attention as a new type of green and sustainable solvent system [8]. NADESs are usually formed from naturally occurring primary metabolites (such as amino acids, sugars, organic acids, or choline derivatives [9]) through a strong intermolecular hydrogen bond network [10]. A notable feature is that they are liquid at room temperature and their melting point is much lower than that of their single components [8,11]. Compared with traditional organic solvents, NADESs not only have advantages such as low volatility, low toxicity, good biocompatibility, and biodegradability [9], but research also reveals that they show excellent solubilizing ability for a variety of insoluble bioactive components, including phenols, flavonoids, and terpenoids [12]. This solubilization effect is believed to mainly result from the strong hydrogen bond interaction formed between solute molecules and NADES components, as well as the unique polar microenvironment of the solvent itself, thereby significantly enhancing the solubility and potential biological efficacy of the active ingredient in food or drug systems [12].

It is particularly notable that the concept of Hydrophobic Natural Deep Eutectic Solvents (HDESs) has been proposed. This type of solvent is composed of natural hydrophobic components (such as medium- and long-chain fatty acids, terpenes, and phytosterols, etc.). Compared with hydrophilic NADESs, HDESs show unique advantages in dissolving and delivering hydrophobic plant active ingredients (such as pentacyclic triterpenoids) [13]. Preliminary research indicates that HDESs not only effectively dissolve highly hydrophobic molecules but may also alter the physical state of active ingredients (such as inhibiting crystallization and forming amorphous forms) or influence their behavior during digestion, potentially improving oral bioaccessibility and bioavailability [13].

Based on this, this study focuses on constructing a menthol–fatty acid hydrophobic eutrophic solvent (HDES) system to systematically explore its mechanism of enhancing the oral bioavailability of OA. For this purpose, HDESs with different fatty acid chain lengths were prepared and characterized for their key physical properties and OA solubility. The intermolecular interactions within the HDES-OA system and the corresponding alterations in the crystalline state of OA were analyzed by FTIR and XRD, respectively. Furthermore, the effects of HDES on the bioaccessibility and intestinal permeability of OA were comprehensively evaluated through in vitro simulated digestion models and Caco-2 cell monolayer models. This work aims to elucidate the structure–activity relationship of fatty acid chain length in HDES-facilitated OA delivery, providing a novel, tunable strategy for the efficient oral delivery of hydrophobic bioactive compounds and expanding the application of HDESs in functional food formulations.

## 2. Materials and Methods

### 2.1. Materials

Oleanolic acid (OA, ≥98%, HPLC) was purchased from Shanxi Jinkangtai Biotechnology Co., Ltd. (Xian, China) and used directly without purification. Menthol (≥95%) was purchased from Wuhan Lanabai Pharmaceutical and Chemical Company (Wuhan, China). n-Octanoic acid (≥99%), decanoic acid (≥99%), genistein (≥98%), and amiloride (≥98%) were purchased from Shanghai Aladdin Biochemical Technology Co., Ltd. (Shanghai, China). Lauric acid (≥98%), oleic acid (analytical grade), fluorescein sodium salt (analytical grade), indomethacin (≥99%), and chlorpromazine (≥98%) were purchased from Shanghai Maclean Biochemical Technology Co., Ltd. (Shanghai, China). Anhydrous ethanol (99.7%), sodium chloride, and calcium chloride (all analytical grade) were, respectively, purchased from Hunan Huihong Reagent Company and Sinopharm Reagent Company (Changsha, China). Methanol (HPLC grade) was purchased from Hubei Feidun Biochemical Technology Co., Ltd. (Wuhan, China). Phosphoric acid (≥85%), sodium hydroxide standard solution, and hydrochloric acid standard solution were, respectively, purchased from Tianjin Comiou Company (Tianjin, China) and Shenzhen Bolinda Company (Shenzhen, China). Bovine bile fluid, pepsin (≥250 U/mg), pancreatin (P7545, 8USP), and lipase (L3126, porcine source, 100–500 U/mg) were purchased from Sigma-Aldrich (St. Louis, MO, USA). Caco-2 cells were obtained from Nanjing Kebai Biotechnology Co., Ltd. (Nanjing, China). Fetal bovine serum, MEM, PBS (pH 7.4), and HBSS (pH 6.8) were purchased from Biological Industries (Shanghai, China). The 0.25% pancreatic enzyme-EDTA and CCK-8 kits (enhanced type) were, respectively, purchased from Biyuntian Company (Shanghai, China) and Beijing Lanjieke Company (Beijing, China).

### 2.2. The Preparation of HDESs

According to the method of Peng Xiao et al., hydrogen bond acceptor (HBA) and hydrogen bond donor (HBD) were mixed in the molar ratio shown in Table 1, placed in the reagent bottle, and stirred for 60 min at a certain rotational speed using a heat-collecting constant-temperature magnetic stirring bath at 80 °C to form a uniform liquid, which was then placed at a dry room temperature for standby [14].

### 2.3. Viscosity Determination of HDESs

Referring to the method of Ahmad et al., the viscosity of all HDESs was measured at room temperature 30 °C and normal pressure using the Kinexus advanced rotational rheometer (Malvern Panalytical, Kinexus pro+, Malvern, UK) with a gap of 0.1 mm and a shear stress of 1 Pa [15]. After the shear viscosity stabilizes, the detection is stopped, and the data is the average of the six data after stabilization.

### 2.4. Determination of the Solubility of OA

Based on the method slightly modified by Silva et al., the solubility of OA in seven types of HDESs was determined [16]. Excess OA was mixed evenly in different HDESs. At 60 °C, the mixture was stirred for 30 min under a constant-temperature magnetic stirrer (at least three independent samples were prepared for the determination of solubility values and standard deviations), with a rotational speed of 300 rpm. After saturation (no further increase after longer mixing), it was centrifuged at 5550× *g* for 10 min using a high-speed centrifuge. The supernatant was separated from the undissolved OA. A 50 µL aliquot of the supernatant was diluted with 4950 µL of chromatographic-grade methanol, filtered through a 0.22 µm membrane, and then analyzed by HPLC using an Agilent HPLC system. This system is equipped with a quaternary solvent delivery system, an autosampler, and a diode array detector (DAD), with a detection wavelength of 210 nm. The separation was performed on an YMC-Pack ODS-A (250 × 4.60 mm) chromatographic column. The column oven and the autosampler are maintained at 30 °C. The mobile phase consisted of chromatographic-grade methanol and 0.1% phosphoric acid (volume ratio of 92.5%:7.5%), and the flow rate was 1 mL/min. The formula for calculating solubility is as follows:

Solubility (mg/mL) = (C_solubility_ × D)/1000 (1)

In the formula, C_solubility_ represents the concentration of OA dissolved by the tested HDES. “D” represents the dilution factor of the sample; 1000 represents the unit conversion.

### 2.5. Preparation of HDES-OA

A 5 mL aliquot of HDESs composed of different HBA and HBD was weighed, and OA was dissolved according to the solubility in the traditional organic solvent ethanol. At 60 °C, the mixture was vortexed using a magnetic stirrer for 1 h to obtain HDES-OA.

### 2.6. In Vitro Digestion of HDES-OA

#### 2.6.1. Oral Phase

Based on the method of Brodkorb et al. with a slight modification, the coordinated static in vitro digestion model proposed by INFOGEST was adopted to study the bioaccessibility of Ethanol-OA and HDES-OA [17]. Portions of 5 mL of Ethanol-OA and HDES-OA were, respectively, mixed with 4 mL of NaCl (2 M) solution, followed by the addition of 30 µL of 0.3 M CaCl_2_ and 970 µL of distilled water, giving the final volume of 10 mL. The mixture was incubated at 37 °C for 2 min.

#### 2.6.2. Gastric Phase

The 10 mL of oral phase solution was mixed with 8 mL of NaCl solution, 5 µL of CaCl_2_ was added, and the pH was adjusted to 3.0 with 1.0 M HCl to prepare simulated gastric fluid (SGF). Disperse pepsin (≥250 U/mg) was dispersed in SGF with 1950 µL of distilled water, resulting in a final volume of 20 mL. The mixture was incubated at 37 °C with stirring (200 rpm) for 2 h.

#### 2.6.3. Intestinal Phase

A 20 mL aliquot of the gastric sample was placed in a clean beaker and incubated in a 37 °C water bath for 10 min. The NaCl solution was preheated in a 37 °C water bath, then 8 mL of electrolyte stock solution was added and the pH was adjusted to 7 with NaOH solution (0.05–1 M) [18]. A 3 mL aliquot of bovine bile fluid (10 mM), containing pancreatin (P7545, 8USP) and lipase (L3126, porcine source, 100–500 U/mg) was stirred into the 20 mL of chyme. The resulting mixture was adjusted to pH 7. Then, 40 µL of CaCl_2_ solution (0.3 M) was added and the pH readjusted to 7.0 [19]. The resulting mixture was incubated at 37 °C and 200 rpm for 2 h. During digestion, 1 M NaOH standard solution was added to maintain the pH at 7.0, and the volume of NaOH consumed was recorded at different digestion times.

### 2.7. Determination of the Biological Accessibility of HDES-OA

Based on the method slightly modified by Martin et al., the bioaccessibility of OA in the digested samples was determined [20]. After digestion was completed, the sample was centrifuged at 8000 rpm for 10 min. The intermediate micelles were collected. A 2 mL aliquot of the sample was mixed with 6 mL of anhydrous ethanol by vortexing for 30 min at room temperature and was then allowed to stand. Then, 1 mL of the supernatant was filtered through a 0.22 µm membrane, and the OA content in the micelles was determined by HPLC. Each sample should be analyzed at least three times. The formula for calculating the bioaccessibility of OA is as follows:

Bioaccessibility % = C_Micelle_/C_Digesta_ × 100% (2)

In the formula, C_Micelle_ represents the concentration of OA in the micelle fraction, and C_Digesta_ represents the concentration of OA in the entire sample after the pH-stat experiment.

### 2.8. Characterization of OA Treated by HDES

#### 2.8.1. FTIR Spectral Analysis

Samples were prepared using the KBr pellet method and scanned in the wavenumber range of 4000–400 cm^−1^ using an FTIR (Nicolet-iS 5, Thermo Fisher, Waltham, MA, USA), with the resolution adjusted to 4 cm^−1^ [21].

#### 2.8.2. X-Ray Diffraction Analysis

The crystalline form of the sample was analyzed using X-ray diffraction (XRD). The instrument settings were as follows: a copper target was used as the radiation source with a wavelength of 1.5401 Å, the working voltage was set at 40 kV, the working current was 40 mA, the scanning step was 0.02°, the scanning rate was 5°/min, the divergence and anti-reflection slit was 1.0 mm, a receiving slit was 0.1 mm, and the scanning range was 5.0–35.0° in 2θ [22].

### 2.9. Construction of the Caco-2 Cell Model

#### 2.9.1. Cell Culture

Based on a slight modification by Ojeda-Serna et al., Caco-2 cells were grown in MEM medium supplemented with 20% fetal bovine serum (FBS), with a final pH of 7.2–7.4, and incubated at 37 °C, 5% CO_2_, and 95% air in a humidified incubator [23]. During this period, the culture medium was changed every day until the cells reached 80–90% aggregation. Then, they were digested with trypsin for 2.5 min and subcultured [24].

#### 2.9.2. Cytotoxicity Determination

To determine the appropriate concentration of the digestive micellar phase in cell experiments, its in vitro cytotoxicity was evaluated based on the CCK-8 method [25]. The experiment was set up with a blank control group, a positive control group and an experimental group. The blank control group only added MEM; the positive control group was added with cell suspension. The experimental group was added with cell suspension and HDES-OA. The specific operation is to inoculate 100 µL of Caco-2 cells into a 96-well microplate, maintain a density of 1 × 10^5^ cells/mL, and incubate at 37 °C for 24 h to allow for cell attachment. Then, the complete culture medium was removed. A 100 µL aliquot of the digested substances, diluted with the culture medium in a series of concentration gradients (filtered through a 0.22 µm membrane), was added to the experimental group. The positive control group received fresh culture medium, and the blank control group received MEM only. After 24 h of incubation, 10 µL of cck8 solution was added and incubation continued in a CO_2_ incubator for 0.5 to 4 h. Then, the absorbance values at 450 nm were measured using a microplate reader, and the cell viability (Viability %) was calculated according to Equation (3).

Viability % = (OD_T_ − OD_K_)/(OD_C_ − OD_K_) × 100% (3)

In the formula, OD_T_ represents the absorbance value of the experimental group. OD_K_ represents the absorbance value of the blank group. OD_C_ represents the absorbance value of the positive control group.

#### 2.9.3. Establishment of Caco-2 Cell Monolayer

The method for establishing the cell model was adapted from Liang et al., with slight modifications [26]. The well-growing Caco-2 cells in the logarithmic phase were washed twice with PBS. After digestion with about 1 mL of trypsin, 4 mL of complete culture medium was added to terminate the digestion, and the cells were repeatedly pipetted to ensure a single-cell suspension. The cell number was determined under a 100× inverted microscope using a hemocytometer. A cell suspension with a cell concentration of 1 × 10^5^ cells/mL was prepared by diluting with MEM medium and then inoculated onto a collagen-coated polytetrafluoroethylene membrane with a pore size of 0.4 µm and a diameter of 1.12 cm^2^ in a 12-well Transwell plate. A 0.5 mL aliquot of cell suspension was added to the apical (AP) and 1.5 mL of culture medium was added to the basolateral (BL) side. The medium was changed the next day. It was changed every other day for the following week. After the second week, it was changed daily. The cells were fully differentiated after about 21 days of culture.

#### 2.9.4. Caco-2 Measurement of Cell Transepithelial Electrical Resistance (TEER)

The transmembrane resistance of the cultured Caco-2 cells was monitored (0–21 days). Before measurement, the electrodes were sterilized by soaking them in 75% alcohol for 15 min, then taken out and air-dried, and finally rinsed with HBSS. The culture medium was aspirated from the AP side and BL side of the Transwell plate, and the cell layer was washed 1 to 2 times with HBSS. Subsequently, 0.5 mL of HBSS was added to the AP side and 1.5 mL to the BL side. For measurement, the short end of the electrode was inserted vertically into the AP side of the culture chamber (avoiding contact with the cell monolayer), and the long end was immersed in the BL side. After the reading stabilized, the value was recorded. Measurements were taken every three days during the cultivation period. TEER was calculated as follows:

*TEER* = (Ω − Ω_0_) × 1.12 (4)

In the equation, TEER is the transepithelial electrical resistance (Ω·cm^2^); Ω is the resistance of the cell monolayer (Ω); Ω_0_ is the resistance of the blank insert without cells (Ω); and A is the effective membrane area of the 12-well Transwell plate (1.12 cm^2^).

#### 2.9.5. Measurement of the Apparent Permeability Coefficient (Papp)

Before the transport experiment, the differentiated Caco-2 monolayer was washed three times with HBSS preheated at 37 °C. After the last wash, it was equilibrated in the incubator for 30 min. The integrity of the intestinal epithelial barrier was evaluated by referring to the method of Li et al. [27]. A 0.5 mL aliquot of sodium fluorescein solution in HBSS was added to the AP side, and 1.5 mL of HBSS solution was added to the BL side. The samples were incubated in an incubator for 120 min. During this period, a 100 µL sample solution was taken from the BL side every 30 min for determination, and 100 µL of HBSS solution was added to replenish the volume. Sodium fluorescein standard solutions (0.25, 0.5, 1, 2, 4, and 8 µg/mL) in HBSS were prepared. Their absorbance was measured at 490 nm using a microplate reader, and a standard curve was plotted (concentration vs. absorbance). The apparent permeability coefficient (Papp) of sodium fluorescein was calculated using Equation (5) to assess membrane permeability [28].

(5) $$Papp=\frac{△Q}{△t}\times \frac{1}{A\times C_{0}}$$

where Papp is the apparent permeability coefficient (cm/s); A is the surface area of the membrane (cm^2^); C_0_ is the initial concentration of sodium fluorescein on the AP side (ug/mL); and ΔQ/Δt is the transport rate of sodium fluorescein (µg/s).

#### 2.9.6. Cell Uptake

Based on the method of Ma et al., Caco-2 cells were inoculated in 12-well plates at a density of 1 × 10^5^ cells per well [29]. During the first week, the medium was changed every two days, and daily during the second week. After approximately 14 days of culture, the complete medium was removed. The cells were rinsed three times with PBS and then equilibrated in the incubator for 30 min in buffer. The absorption of OA by cells was tested by adding several diluted OA digestion micellar solutions (600 µL) to the cells and incubating them for 4 h. After incubation, the cell monolayer was washed three times with PBS to remove free samples. Then, 200 µL of cell lysis buffer was added. The cell suspension was collected and subjected to ultrasonic treatment and centrifuged (13,500 rpm for 10 min) to obtain the supernatant. OA was extracted from the supernatant with anhydrous ethanol and quantified by HPLC. The cellular uptake rate was calculated as follows:

Uptake % = M_cell_/M_micelle_ (6)

where M_cell_ is the amount of OA taken up by the cells and M_micelle_ is the amount of OA in the applied micellar phase.

#### 2.9.7. The Absorption Pathways of Cells

Caco-2 cells were cultured in 12-well plates for 14 days, and the monolayer was washed three times with preheated PBS. Media containing specific inhibitors (namely 100 μmol·L^−1^ genistein, 300 μmol·L^−1^ indomethacin, 50 μmol·L^−1^ amiloride, 31 μmol·L^−1^ chlorpromazine, and the combined application of 100 μmol·L^−1^ genistein with 300 μmol·L^−1^ indomethacin [30,31]) were added, and the cells were incubated for 1 h. Then, the medium was aspirated and the digested micellate phase diluted with MEM was added. Incubation continued for 2 h. Subsequently, the cell monolayer was washed three times with PBS. After adding the cell lysate, the supernatant was obtained by centrifugation. OA was extracted with anhydrous ethanol, quantified by HPLC, and the cellular uptake rate was calculated.

#### 2.9.8. Cell Transport

Caco-2 cells were seeded in 12-well plates at a density of 1 × 10^5^ cells per well. The medium was changed every two days during the first week and daily during the second week [32,33]. After 21 days of culture, the monolayers were washed three times with HBSS and equilibrated for 30 min. For transport in the apical-to-basolateral (AP-to-BL) direction, a 0.5 mL aliquot of diluted OA-containing micelles was added to the AP side, and 1.5 mL of blank HBSS was added to the BL side. At specified times (0.5, 1, 2 and 4 h), a 200 µL sample was collected from the BL side and replaced with an equal volume of fresh HBSS. OA was extracted from the samples with anhydrous ethanol and quantified by HPLC. The transport (secretion) percentage was calculated as follows [29].

Secretion % = M_Bas_/M_initial_ ×100% (7)

where M_Bas_ is the content of OA in the BL solution, and M_intial_ is the initial amount of OA on the AP side before transport.

### 2.10. Determination of Bioavailability

Based on the cellular uptake and transport results, the total cellular absorption rate of OA was calculated using Equation (8) [29]. The bioavailability of OA was expressed as the product of the total cell absorption rate and bioaccessibility, as shown in Equation (9) [29].

Cell absorption % = Uptake % + Secretion %(8)

Total bioavailability% = Cell absorption% × Bioaccessibility%(9)

### 2.11. Statistical Analysis

Based on the results of three repeated calculations, they were expressed as the mean ± standard deviation. A one-way analysis of variance (ANOVA) followed by Duncan’s multi-range test was performed using the SPSS version 27.0 (IBM, Almonk, NY, USA) to determine significant differences among groups. A *p*-value < 0.05 was considered statistically significant.

## 3. Results and Discussion

### 3.1. The Influence of HDESs on the Bioaccessibility of OA and Its Physical Property Basis

**Figure 1:**
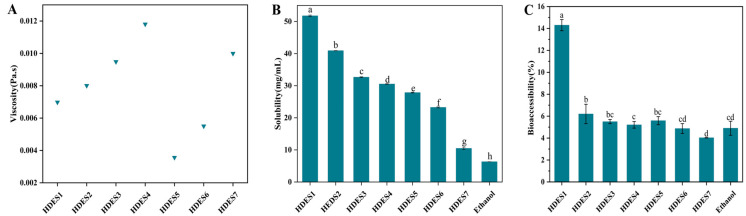
Viscosity of different HDESs at room temperature, 30 °C, and atmospheric pressure (**A**). The solubility of OA in HDESs composed of different types of HBA and HBD at 60 °C (**B**). The bioaccessibility of OA in HDESs composed of different components after oral gastrointestinal digestion (**C**). The data in the figure were expressed as mean ± standard deviation (mean ± SD). Different lowercase letters represent significant differences between groups (*p* < 0.05), and the same letter indicates no significant difference between groups.

Bioaccessibility (referring to the proportion of active ingredients that can be released and absorbed during digestion [34]) directly determines the final bioavailability of OA. The in vitro digestion experiments (Figure 1C) show that when ethanol was used as the carrier, the bioaccessibility of OA was 4.90 ± 0.64%, while menthol-based HDESs (HDES1–HDES4) could significantly increase this value to a range of 5.21% to 14.30%. Moreover, with the increase in fatty acid chain length (octanoic acid → oleic acid), it shows a systematic decreasing trend (HDES1: 14.30 ± 0.51% > HDES2: 6.20 ± 0.89% > HDES3: 5.50 ± 0.19% > HDES4: 5.21 ± 0.30%). Although laurate-based HDESs (HDES5–HDES7) had a similar chain-length effect (HDES5: 5.59 ± 0.37% > HDES6: 4.87 ± 0.45% > HDES7: 4.03 ± 0.02%), only HDES5 was significantly superior to the ethanol control (*p* < 0.05). There was no statistically significant difference between HDES6/7 and the control group (*p* > 0.05). These results indicate that the bioaccessibility of OA in HDES vectors has a significant dependence on fatty acid chain length.

The chain-length dependence of bioaccessibility stems from the synergistic effect of the physical parameters of HDESs: in terms of solubility (Figure 1B), the solubility of all HDESs for OA was significantly higher than that of ethanol. However, when the hydrogen bond acceptor (menthol/lauric acid) was fixed, the solubility systematically decreased with the increase in fatty acid chain length—among which, HDES1 (menthol/octanoic acid) showed the best solubility due to the polarity matching of octanoic acid and OA. Long-chain fatty acids have a reduced dissolution efficiency due to steric hindrance, which inhibits molecular interactions. In terms of viscosity (Figure 1A), most of the DESs reported so far exhibit typical high-viscosity characteristics (>100 mPa·s) at 30 °C [35], but the seven HDESs in this study have relatively low viscosity, and the viscosity increases significantly with the increase in chain length under the same hydrogen bond acceptor (e.g., a 69.20% increase from HDES1 to HDES4. Specific values: HDES1: 0.00698 mPa·s; HDES2: 0.00801 mPa·s; HDES3: 0.00949 mPa·s; HDES4: 0.01181 mPa·s). This trend is consistent with the viscosity-regulation mechanism dominated by hydrogen bond networks and van der Waals forces [36], that is, long carbon chains enhance the molecular stacking effect and weaken the fluidity of the system. This systematic viscosity increase may thus suggest a physical explanation for the lack of further bioaccessibility improvement in the longer-chain HDESs (e.g., HDES5–7), which is consistent with the overall performance trend.

Based on the data from this study, we speculate that an increase in the chain length of fatty acids may impede the molecular diffusion process of OA by enhancing the viscosity of the HDES system, thereby reducing its apparent solubility in the carrier. This potential negative correlation between viscosity and solubility may further inhibit the release efficiency of OA during digestion, ultimately tending to lead to a decreasing trend in biological accessibility with increasing chain length.

### 3.2. Characterization of OA After HDES Processing

**Figure 2:**
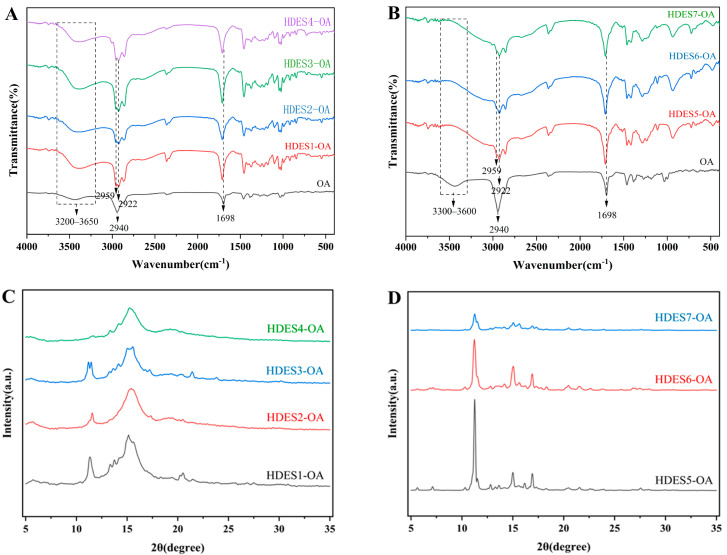
FTIR of HDES-OA (HDES1–4) blends (**A**). FTIR of HDES-OA (HDES5–7) blends (**B**). XRD of OA after HDES1–4 treatment (**C**). XRD of OA treated with HDES5–7 (**D**).

FTIR spectroscopy (Figure 2A,B) revealed that the carbonyl characteristic peak of OA (~1698 cm^−1^) in the HDES-OA system shifted to higher wavenumbers, suggesting molecular interactions (e.g., potential hydrogen bond formation) between OA and HDES components. These interactions may affect the dispersion state of OA by restricting molecular vibration freedom [21,37]. Meanwhile, the -CH_2_ stretching vibration peak (2940 cm^−1^) showed bimodal differentiation at 2959/2922 cm^−1^, indicating a reconfigured hydrophobic microenvironment [38]. It is notable that the intensity of the hydroxyl vibration region (3200–3650 cm^−1^) was significantly enhanced in the menthol HDESs (HDES1–HDES4), while no similar phenomenon was observed in the lauric acid system (HDES5–HDES7). Combining this with the bioaccessibility data (HDES1–HDES4: 14.30–5.21% vs. HDES5–HDES7: 5.59–4.03%), we hypothesize that the enhanced hydroxyl interaction in the menthol carrier or by constructing a stable molecular interaction network, on the one hand, maintains the solubility stability of OA (consistent with the solubility trend in Section 3.1), and on the other hand, promotes the release efficiency during digestion, thereby partially explaining its significantly improved bioaccessibility. In contrast, the absence of hydroxyl groups in the laurate-based systems may weaken carrier–solute compatibility, resulting in a limited improvement in bioaccessibility.

**Figure 3:**
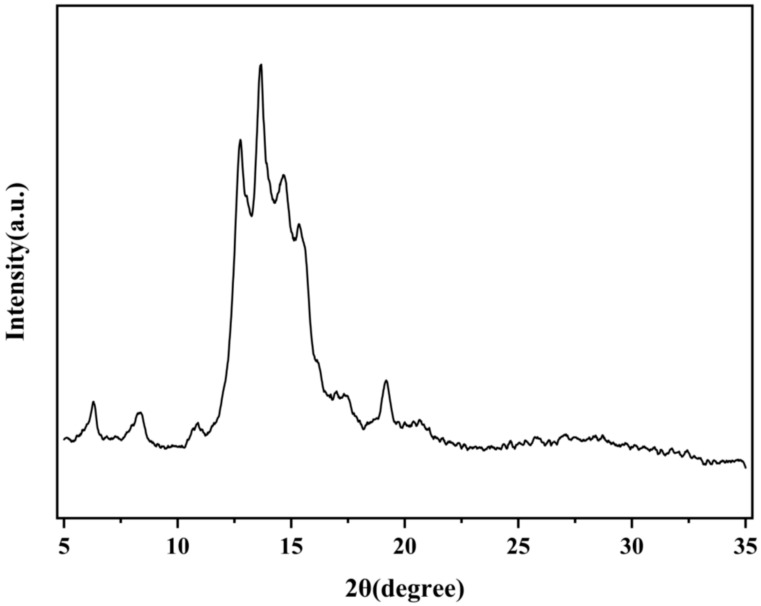
XRD pattern of OA.

X-ray diffraction (XRD), as a core technology for crystal structure analysis, can precisely characterize the crystalline state, crystal form transformation and amorphous degree of substances through the changes in diffraction peak position, intensity, and peak shape. In this study, the effects of different HDES treatments on the crystal structure of OA were analyzed by XRD (Figure 2C,D and Figure 3). The XRD pattern of untreated OA (Figure 3) shows sharp diffraction peaks (such as 2θ = 13.1°, 13.9°, 15.4°, 19.5°), indicating that it has a highly ordered crystal structure [21]. After treatment with HDES1–4 (Figure 2C), the intensity of the diffraction peaks of OA was significantly reduced, but some characteristic peaks were still retained, indicating that the treatment with HDES1–4 partially disrupted the crystal order of OA but did not completely eliminate its crystal structure. After being treated with HDES5–7 (Figure 2D), OA exhibits more significant amorphous characteristics. Particularly, the OA spectrum treated with HDES7 shows broad “steamed bun peaks”, and the original diffraction peaks completely disappear, indicating that the crystal structure of OA has been thoroughly destroyed.

It is worth noting that the completely amorphous HDES5–7 groups (with HDES7 being particularly notable) did not show the expected improvement in bioaccessibility (actual value: 5.59–4.03%) but were significantly lower than the HDES1–4 groups that partially retained crystal orderliness (14.30–5.21%). The reason for this difference between HDES5–7 and HDES1–4 might be that HDES5–7 does not contain menthol in its composition. Therefore, we speculate that the possible reason for this phenomenon is that the limited crystal order retained in HDES1–4 may regulate the sustainably released kinetics of OA by forming a stable molecular interaction network (such as the hydroxyl enhancement effect 5 revealed by FTIR)with solvent components (such as menthol), thus avoiding sudden release and molecular aggregation during digestion. In addition, although HDES5–7 achieves complete amorphous transformation, due to the lack of menthol-mediated molecular compatibility, OA molecules may undergo disordered aggregation due to high energy states, resulting in a reduction in the effective release surface area.

### 3.3. Cell

#### 3.3.1. Cytotoxicity Assessment and Safe Concentration Screening

**Figure 4:**
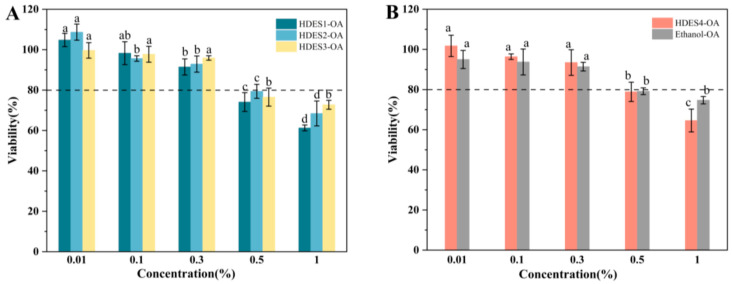
The toxicity of different HDES-OA systems to Caco-2 cells. HDES1-OA, HDES2-OA and HDES3-OA (**A**). HDES4-OA and Ethanol-OA (**B**). The data in the figure were expressed as mean ± standard deviation (mean ± SD). Different lowercase letters represent significant differences between groups (*p* < 0.05), and the same letter indicates no significant difference between groups.

Given that the HDES vector may affect cell viability, in this study, the toxicity of the micellar phase (0.01–1%, *v*/*v*) after in vitro digestion to Caco-2 cells was evaluated by the CCK-8 method to eliminate its interference with subsequent uptake and transport experiments. The results show (Figure 4) that when the micelle concentration is ≥0.5%, the cell survival rate is significantly lower than 80% (the widely accepted toxicity threshold). However, within the concentration range of 0.01–0.3%, the survival rates were all >80%, and there was no statistical difference between the 0.3% and 0.1% concentration treatment groups (*p* > 0.05), suggesting that there was no significant cytotoxicity in this range. According to the principles of toxicological experimental design, the highest concentration (0.3%) that is no different from the low toxic concentration was selected for subsequent transport studies to ensure that the cell activity is not affected by the carrier.

#### 3.3.2. Establishment and Integrity Assessment of Cell Monolayers

**Figure 5:**
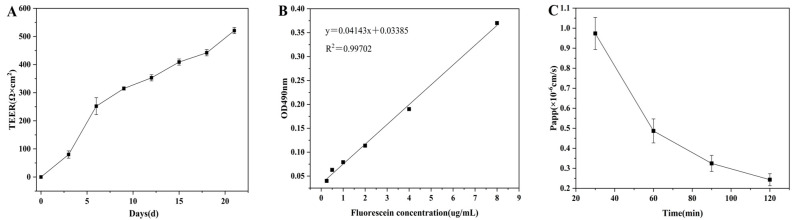
Transmembrane resistance changes in the Caco-2 cell monolayer model within 21 days of culture (**A**). Standard curve of fluorescein sodium (**B**). Papp of fluorescein sodium transport (**C**).

Transmembrane resistance (TEER) monitoring showed that the growth rate of TEER was slow in the early stage of culture (0~3 days) (Figure 5A). From three to six days, it enters the exponential growth phase (reaching 252 Ω·cm^2^), reflecting the active proliferation of cells. A steady upward trend from 6 to 18 days indicates the process of differentiation and integration. By 21 days, the TEER reached 521 Ω·cm^2^, significantly exceeding the monolayer integrity threshold (200–1500 Ω·cm^2^) [39], confirming the formation of a dense differentiation barrier. Permeability verification was accomplished through the apparent permeability coefficient (Papp): based on the standard curve of sodium fluorescein (y = 0.04143x + 0.03385, R^2^ = 0.99702) (Figure 5B), the calculated Papp values were all <1 × 10^−6^ cm/s (Figure 5C), which conforms to the complete monolayer characteristics [27]. Dual indicators confirm that the 21-day culture model has both excellent barrier integrity (TEER > 500 Ω·cm^2^) and standard osmotic regulation ability (Papp < 10^−6^ cm/s), providing a reliable biological interface for transport experiments.

#### 3.3.3. Exploration of the Uptake of OA by Caco-2 Cells and Its Mechanism

##### Cellular Uptake

**Figure 6:**
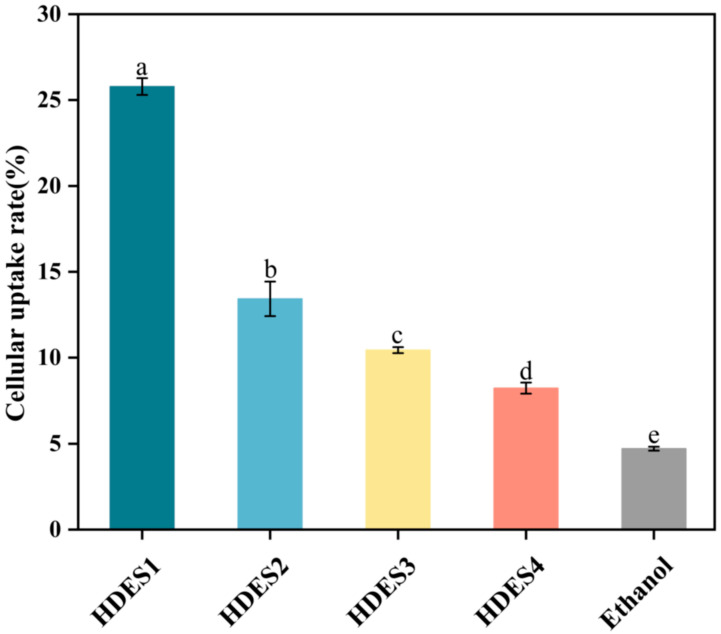
The uptake rate of OA by Caco-2 cells after incubation in different DES systems for 4 h. The data in the figure were expressed as mean ± standard deviation (mean ± SD). There were significant differences between the groups represented by different lowercase letters (*p* < 0.05).

The quantitative determination results of intracellular OA (Figure 6) showed that, compared with the conventional ethanol vector (4.71 ± 0.12%), the HDES system significantly increased the OA uptake efficiency (8.24 ± 0.32%–25.79 ± 0.49%), the relative uptake up to approximately 5.5-fold that of the ethanol system. Notably, a clear chain-length gradient effect was observed within the HDES series: uptake was highest for HDES1 (octanoic acid-based; 25.79 ± 0.49%) and successively decreased through HDES2 (13.43 ± 1.01%) and HDES3 (10.44 ± 0.17%) to the lowest for HDES4 (oleic acid-based; 8.24 ± 0.32%) (*p* < 0.05). This negative correlation trend is highly consistent with the change pattern of bioaccessibility in Section 3.1, indicating that the influence of fatty acid chain length persists throughout the sequential stages of digestion, release, and cellular absorption.

From the perspective of molecular mechanisms, this chain-length effect may stem from the synergistic effect of multiple factors. Firstly, an increase in chain length significantly enhances the viscosity of HDES (as shown in the data in Section 3.1). Theoretically, according to the Stokes–Einstein relation (D = kT/6πηr), higher viscosity could contribute to slower diffusion at the carrier–cell interface, though cellular uptake is ultimately governed by more complex membrane interactions. Secondly, short-chain fatty acids (such as C8-octanoic acid) endow HDES with low hydrophobicity and high polarity, which may facilitate penetration of the aqueous hydration layer and promote initial adsorption to the phospholipid bilayer, in line with the general “like-dissolves-like” principle. Furthermore, the steric hindrance effect of long-chain fatty acids (such as C18 oleic acid) could interfere with lipid raft-mediated transport pathways—a possibility supported by literature reports on the chain-length selectivity of fatty acid transporters (FATPs) [36], although direct experimental validation in this study remains for future work.

Overall, the viscosity-mediated diffusion limitation, the hydrophobic-regulated changes in membrane affinity, and the interference of steric hindrance on transmembrane transport jointly constitute the chain-length-dependent uptake rate decline mechanism. This discovery highlights that short-chain HDESs (such as HDES1) can effectively break through the cellular uptake bottleneck of hydrophobic components by synergistically optimizing the ternary characteristics of “low viscosity—moderate hydrophobicity—molecular flexibility”.

##### Cellular Absorption Pathway

**Figure 7:**
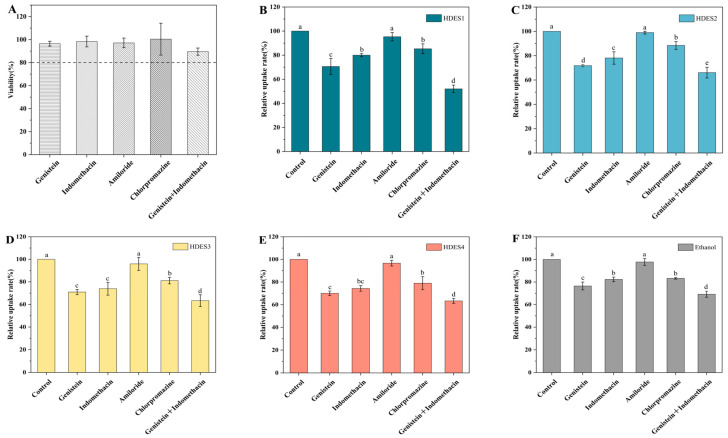
Toxicity of different inhibitors on Caco-2 cells (**A**). Relative uptake rate of OA by Caco-2 cells treated with different inhibitors (**B**–**F**). The data in the figure were expressed as mean ± standard deviation (mean ± SD). Different lowercase letters represent significant differences between groups (*p* < 0.05), and the same letter indicates no significant difference between groups.

To explore the cellular mechanism by which HDES vectors enhance the uptake efficiency of OA (1.75–5.48 times higher than that of the ethanol system), this study selected a series of specific inhibitors: genistein (lipid raft/fossa endocytosis and PTK inhibitor) [30,31], indomethacin (lipid raft/fossa endocytosis and COX inhibitor) [30,31], amiloride (macrocytic drinking inhibitor) [40,41], and chlorpromazine (mesh protein endocytosis inhibitor) [42,43]. The cell survival rates at the experimental concentrations were all above 80% (Figure 7A), ensuring the reliability of the mechanism exploration.

Inhibitor effect analysis revealed (Figure 7B) that genistein or indomethacin alone reduced the uptake rate of OA by 18–30% (inhibitor concentrations and exposure times are detailed in the Methods Section). The inhibitory effect was significantly enhanced when combined (*p* < 0.05), strongly suggesting a substantial contribution of lipid-raft/caveolin-mediated endocytosis—which is consistent with the lipid-raft-dependent transport mechanism reported in the literature. In contrast, there was no statistically significant difference between the amiloride treatment group and the control group (*p* > 0.05), effectively excluding the involvement of the macrocytic drinking pathway. It is worth noting that chlorpromazine treatment led to a significant decrease in uptake rate, confirming the concurrent involvement of clathrin-mediated endocytosis. This observation is consistent with the clathrin-mediated endocytosis model reported in the literature [42,43].

Therefore, the HDES-OA system exhibited cellular uptake through lipid raft/fosprotein-mediated endocytosis (core pathway) and reticin-mediated endocytosis (helper pathway). This dual endocytosis mechanism may be the key basis for HDES vectors to optimize the transmembrane transport efficiency of hydrophobic components. This mechanism is highly synergized with the improvement in bioavailability: the lipid raft pathway can directly avoid lysosomal degradation and maintain the biological activity of OA (Reference [44] reported that caveolin-mediated transport can reduce first-pass metabolism). Meanwhile, the reticin pathway provides supplementary uptake channels, and the two jointly ensure the efficient transmembrane transport of HDES-OA.

#### 3.3.4. Cellular Secretion

**Figure 8:**
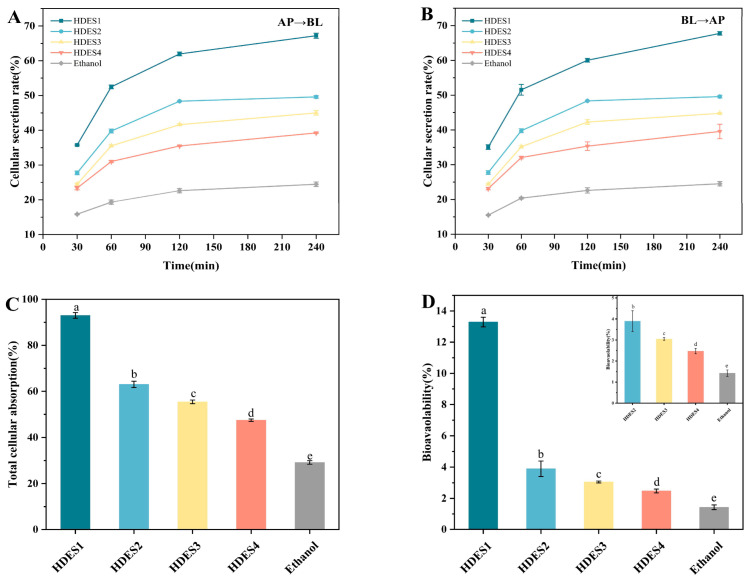
The secretion rate of Caco-2 cells at different incubation times (**A**,**B**). The total absorption rate of OA in Caco-2 cells (**C**). The bioavailability of OA in different HDESs (**D**). The data in the figure were expressed as mean ± standard deviation (mean ± SD). Different lowercase letters represent significant differences between groups (*p* < 0.05).

This study systematically analyzed the regulatory rules of HDES vectors on the transmembrane secretion behavior of OA through a monolayer model of Caco-2 cells (Figure 8A,B). Experimental data show that all HDES systems exhibit significant time-dependent secretion characteristics. Key findings indicate that the secretion rates of all HDES-OA systems significantly exceed those of the ethanol control group (*p* < 0.05) and show a systematic decreasing trend with the increase in fatty acid chain length (HDES1 > HDES2 > HDES3 > HDES4). This rule is highly consistent with the characteristics of decreasing cellular uptake rate mentioned above. It was confirmed that the chain-length effect runs through the entire process of intestinal absorption. From a mechanistic perspective, the high-viscosity property of long-chain HDES may reduce molecular diffusion (as suggested by the Stokes–Einstein relation, D∝1/η), and their increased hydrophobicity could potentially influence interactions with membrane-associated transporters. The combined effect of these factors is hypothesized to contribute to the observed decrease in transport efficiency, though direct validation (e.g., ABC transporter assays) would be required to confirm this. This finding provides a potential design insight for HDES carriers: short-chain systems may enhance the overall intestinal absorption of OA by favoring a balance of diffusion kinetics and membrane interaction.

#### 3.3.5. Total Cell Absorption Rate and Bioavailability

Based on the results of the cell uptake and secretion experiments, this study defined the total cell uptake rate as the sum of the uptake rate and the secretion rate (Figure 8C). As shown in the figure, the DES carrier significantly increased the total absorption rate of OA (all higher than that of the traditional ethanol system), and there were significant differences among different HDESs (*p* < 0.05). The total absorption rate systematically decreases with the increase in the chain length of hydrogen-bond donor fatty acids (HDES1 > HDES2 > HDES3 > HDES4), and this trend is consistent with the chain-length effect of cell uptake rate and secretion rate in the previous text.

According to the standard pharmacokinetic model [44,45], the bioavailability of OA is calculated as the product of bioaccessibility and total absorption rate (characterizing the proportion entering systemic circulation). The bioavailability of the Ethanol-OA system was only 1.43 ± 0.15%, attributed to the dual limitations of its low bioaccessibility (4.90 ± 0.64%) and total absorption rate (29.18 ± 0.76%). The bioavailability of the HDES system significantly exceeded that of the ethanol control group (*p* < 0.05), specifically manifested as HDES1 (13.29 ± 0.31%) > HDES2 (3.90 ± 0.50%) > HDES3 (3.05 ± 0.06%) > HDES4 (2.47 ± 0.12%). The trend of decreasing bioavailability with increasing chain length is highly consistent with the data from digestion experiments (Section 3.1) and cell transport experiments (Section 3.3), confirming that HDES can systematically enhance the bioavailability of OA. The observed trend is consistent with the identified chain-length-dependent variations in key physicochemical properties of the HDES, which collectively influence the overall delivery efficiency.

## 4. Conclusions

This study aims to explore the effect and mechanism of HDES based on menthol and fatty acids of different chain lengths on the in vitro oral bioavailability of OA. The results show that, compared with traditional ethanol carriers, HDES (especially the short-chain fatty acid system HDES1) significantly improves the in vitro bioavailability proxy of OA (up to 9.3 times that of ethanol carriers), and the effect weakens with the increase in fatty acid chain length (HDES1 > HDES2 > HDES3 > HDES4). HDESs collaboratively optimize OA delivery and absorption through a multi-level linkage mechanism: effectively enhancing bioaccessibility in the digestion stage (HDES1: 14.30% vs. ethanol: 4.90%); in the absorption phase, the efficiency of transmembrane transport may be enhanced by activating lipid raft/omentum protein dual-path endocytosis (HDES1 uptake rate: 25.79% vs. ethanol: 4.71%). By revealing a clear fatty acid chain length–activity relationship and suggesting its stagewise contribution to bioavailability enhancement, this work offers insights for the design of tunable delivery systems aimed at improving the absorption of hydrophobic bioactive compounds in functional foods and nutraceuticals.

## Figures and Tables

**Table 1:** The molar ratio composition of HDESs.

Abbreviation	HBA	HBD	Molar Ratio
HDES1	Menthol	n-Octanoic acid	2:1
HDES2		Decanoic acid	2:1
HDES3		Lauric acid	2:1
HDES4		Oleic acid	2:1
HDES5	Lauric acid	n-Octanoic acid	1:2
HDES6		Decanoic acid	1:2
HDES7		Oleic acid	1:2

## Data Availability

The original contributions presented in this study are included in the article. Further inquiries can be directed to the corresponding author.
